# Implementation of a collaborative online international learning program in nursing education: protocol for a mixed methods study

**DOI:** 10.1186/s12912-022-01031-9

**Published:** 2022-09-08

**Authors:** D. Kiegaldie, A. Pepe, L. Shaw, T. Evans

**Affiliations:** 1 Faculty of Health Science, Youth and Community Studies, Holmesglen Institute, 488 South Road, VIC 3189 Moorabbin, Australia; 2 Eastern Health Clinical School, Monash University, VIC 3128 Box Hill, Australia; 3 Healthscope, Holmesglen Private Hospital, 488 South Road, VIC 3189 Moorabbin, Australia; 4 Faculty of Health Sciences, School of Behavioural and Health Science, Australian Catholic University, 115 Victoria Parade, VIC 3065 Fitzroy, Australia; 5 ARCH, School Allied Health, Human Services and Sport, La Trobe University, VIC 3086 Bundoora, Australia; 6 Department of Nursing Education and Health Studies, Northwestern Polytechnic, Grande Prairie, Alberta, Canada

**Keywords:** Education, Nursing, Collaborative online learning, Simulation, Virtual reality, COIL, Cultural awareness

## Abstract

**Background:**

An essential component of becoming a professional nurse is a perspective of global health issues and an awareness of diverse populations. Collaborative online international learning (COIL) using digital technologies, offers meaningful and rewarding opportunities to develop international partnerships between nurses from other countries, without economic, organisational or geographical barriers. Despite reported advantages of using COIL, few COIL interventions have been identified in the nursing literature. The aims of this study are to develop, implement and evaluate a COIL program between Australian and Canadian pre-registration nursing students.

**Methods:**

The study will utilize a mixed methods approach incorporating pre and post-test surveys, focus groups, and semi-structured interviews of key stakeholders. The design will adhere to The State University of New York (SUNY) COIL’s criteria for intercultural/international learning opportunities. Participants will be recruited from nursing programs at an Australian Training and Further Education Institute and a Canadian college. Bennett’s stages of intercultural competence will provide the theoretical framework for the research. Four specific research interventions will be developed for this project. For students, there will be an online virtual community to allow students and teachers to communicate, socially connect and share resources with each other. Virtual reality simulations will be employed within a virtual global classroom to promote collaborative, intercultural learning. For faculty, a virtual community of practice will provide a platform for faculty to share education and research ideas and participate in collaborate research opportunities.

**Discussion:**

This study will evaluate the outcomes of a nursing COIL program. It will measure participants’ views on COIL, its contribution to student learning, changes in cultural awareness, organisational impact and research productivity. It will provide nursing students with the opportunity to become global leaders in nursing care and for faculty to develop international research skills and outputs. The findings from the study will allow further refinement of future nursing COIL programs.

## Background

The complexity of globalisation, health issues that go beyond national borders, and the challenges associated with rapid geopolitical change, highlight the importance of preparing the future nursing workforce for global healthcare challenges [1]. The opportunity to foster an international perspective is now considered a cornerstone in nursing education [2, 3]. The World Health Organization’s nurse educator core competencies acknowledge the changing face of nursing education and practice. They advocate for the nursing profession to practice, lead and adapt to new evidence and increasingly diverse populations [4].

Given the impact of COVID-19 on the international healthcare education landscape, the value of building and sustaining international connections has never been more important [5, 6]. Further, the increased use of collaborative digital learning technologies, such as cloud-based communication platforms and virtual reality, presents additional opportunities to deepen global engagement of place-bound students and educators [7–10]. The COIL model, pioneered by the State University of New York (SUNY) [11] offers meaningful and rewarding opportunities for learners to engage with international partners across borders, regardless of personal constraints or economic challenges [9, 12]. The premise of a COIL approach is that the shared syllabus is both co-created and co-taught by two or more faculties from different nations, emphasizing experiential and collaborative student learning [12]. Students engage in highly interactive and shared problem-solving exercises with international peers [13]. COIL courses have a determined timeframe where students of two or more faculties engage in shared course content, communicate, and share their ideas and thoughts [12].

Whilst there are limited studies of COIL programs in nursing, findings indicate that COIL can facilitate partnerships, increase cultural competence and prepare nurses to work collaboratively with nurses from other cultures [9, 12, 14]. It has also been shown to increase global awareness, assist in breaking down the global hierarchy and develop global leadership competencies [9, 13].

This project will establish a COIL program between a Training and Further Education (TAFE) Institute in Victoria, Australia and a post-secondary educational institution in Alberta, Canada. It will foster a global learning and research environment for nursing students and faculty. The COIL program will create multiple online environments to facilitate collaboration, sharing of resources, and the development and implementation of novel educational and academic experiences for students and faculty. Four specific research interventions will be developed for this project: 1. Online Virtual Community, 2. Virtual Reality/Virtual Simulation, 3. Virtual Global Classroom, 4. Virtual Community of Practice. The aims of the project are to develop and implement collaborative online environments that: (i) Deepen global engagement of place bound students and faculty, (ii) Enable students and faculty to work collaboratively to address globally relevant issues, (iii) Facilitate an exploration of self and promote cultural awareness, (iv) Foster an appreciation of cultural diversity, (v) Promote international education and research collaboration.

## Methods and study design

### Design

The study design will incorporate multiple approaches to address the research questions using a mixed—methods approach. Bennett’s stages of intercultural competence [15] will provide the underpinning theoretical framework for the research. Bennett (2004) defines intercultural learning as acquiring increased awareness of subjective cultural context (world view), including one’s own, and developing greater ability to interact sensitively and competently across cultural contexts as both an immediate and long-term effect of exchange [15]. In the context of this project the cultural exchange will be virtual.

Using pre and post-test surveys, quantitative measures will measure growth in students’ interpersonal communication skills, adaptability, confidence, cultural awareness, perspectives on differences and similarities of nursing practices, peer collaboration and the effect of the teaching and learning interventions. A post COIL survey for faculty will measure their views on the COIL teaching experience and perceptions of participating in research activities in the Virtual Community of Practice. Qualitative measures will focus on gaining further insight into the COIL experience from the perspective of all participants, including students, faculty, and the project team.

### Participants and recruitment methods

#### Students

The COIL project will recruit eligible student participants from the Diploma and Bachelor of Nursing at the Australian TAFE Institute and the Bachelor of Science in Nursing degree program at the Canadian post-secondary educational institution (Table 1). Eligible students are those who have completed or are undertaking subjects relevant to the COIL subject matter.

**Table 1:** Eligible students and numbers included

Course name	Course description	Number of students (approximately)
Australian Bachelor of Nursing (BN)	3-year undergraduate degree	200 from all 3 years of the course
Australian Diploma of Nursing (DN)	2-year course run over 4 stages	300 students
Canadian Bachelor of Science in Nursing (BScN)	4-year BScN	100

#### Allocation of students to the learning activities

Students will be invited to participate in a range of COIL educational experiences. Participation in the COIL program will be voluntary and considered an extra-curricular activity. It will not be included as a core teaching or assessment requirement within the nursing courses. However, the learning opportunity will be promoted to students as innovative, highly relevant, and professionally beneficial for volunteering students.

#### Faculty staff members

Faculty staff will work with the research staff to develop lesson plans and educational materials for the COIL project. There will be approximately 10 teachers involved in the development and delivery. Teachers will mentor and coordinate students throughout the duration of the COIL experience and receive training on how to facilitate discussions and student learning. Faculty staff will be invited to participate in the Virtual Community of Practice where they can collaborate on teaching and research activities.

#### Student and faculty recruitment

The research study will involve recruitment of a convenience sample of consenting students. Following advertisement of the COIL program, students and faculty will be invited to participate in the research via an email sent from each organisation’s faculty office. The participant information will be attached to the email. Students and faculty will be advised they can choose to not participate in the study without any negative consequences. Within the email, they will be asked to click on a link which will take them to an online survey. Students or faculty can then mark a checkbox in the survey to indicate their consent to participate in the survey(s) and to participate in an audio-recorded focus group interview.

#### Project team: Faculty staff, Project Managers and Marketing Managers

Several teachers (*n* = 6) will be directly involved in designing two new Modules. Marketing Managers (*n* = 2) will be involved in promoting the project at a local, national and international level. Project managers (*n* = 4) from both countries will be involved in setting up each stage of the project and ensuring key milestones are reached. All relevant personnel involved in these activities will be invited to participate in a focus group interview to explore their views and experiences of the development and implementation process (and marketing success from the marketing team) of the COIL project.

#### Project team recruitment and consent

Project team members will be recruited to join a focus group interview via an email with the participant information attached. Faculty will be advised they can choose to not participate in the study without any negative consequences. The email will include a link where project team members can mark a checkbox to indicate their consent.

### Setting

All activities relating to this project will be online using Microsoft Teams™ and Zoom™. Online activities will take place in the institutions’ libraries, classrooms, or at students’ homes depending on current government and institutional COVID-19 restrictions. The teacher training session and the Virtual Global Classroom will run online at a time suitable for live synchronous international collaboration between Australia and Canada.

### Interventions

#### Online Virtual Community (OVC)

Using several channels created in Microsoft Teams™, the OVC will provide a virtual platform where students and teachers can communicate and socially connect with each other. Key features of the OVC will include orientation materials, educational resources, information on the educational activities including modules, chat forums (students and teachers), COIL calendar.

#### Virtual Reality (VR) / Virtual Simulation (VSim)

Four existing VR scenarios (previously developed by the Australian Institute using a Victorian Government research grant) will be jointly delivered to all nursing students. Through a collaborative content development process, two additional VR/VSim scenarios will be created and implemented into the COIL environment: Indigenous Health (Module 5) and Global Health (Module 6). (Table 2).

**Table 2:** COIL VR modules

Module 1: The Verbally Aggressive Person (existing)
Module 2: The Deteriorating Patient (existing)
Module 3: The Patient with Cognitive Impairment (existing)
Module 4: Palliative and End of Life Care (existing)
Module 5: Indigenous Health (NEW)
Module 6: Global Health (NEW)

The Indigenous Health and Global Health Modules will include content that is already covered as part of the nursing courses. Indigenous experts from each respective country will be consulted to ensure appropriate standards are met. Note*. The topics explored and the use of VR/VSim technology in the following COIL VGC Modules are a fundamental part of the Nursing students’ curricula.

#### VR/VSim technical components

CenarioVR™ is a virtual reality software published by ELB Learning [16]. It allows for student learning of branching and interactive virtual simulation scenarios in a 360-degree learning environment. This software allows users to follow a patient through a clinical scenario in an adaptive learning simulation. This is achieved through pop-up text boxes, questions, feedback, decision points, and reflection tasks. Students will be provided information as to how to access and use the software. Any technological issues that arise will be raised and managed via Microsoft Teams™ channels in the OVC and a technology administrator.

#### Virtual Global Classroom

Each VR Module will be linked with the VGC through Microsoft Teams™ where students will be provided a Zoom™ link to participate in a briefing on the module and a follow up synchronous ‘live’ debriefing to discuss features of the simulation scenario. The debrief will be co-facilitated by teachers from Australia and Canada.

### Stages of engagement in the interventions for students

#### Join the OVC

Students will join the OVC and receive an orientation and information about the COIL project, how to sign up for the VGCs and how to access the Microsoft Teams™ channels. During the week of orientation, students will virtually meet each other, their teachers, gain access to resources and begin to engage in the COIL environment.

#### Engage in the learning experiences

After joining the OVC, students will be asked to complete several activities. This includes engaging with the VR/VSim module(s) independently at their own time, place and venue.

#### Attend the VGCs

Students will attend their scheduled 2-h virtual global class co-hosted by teaching staff at both organisations to learn with their international peers, debrief about the virtual simulation modules and engage in several interactive learning activities using Zoom™. This will provide an opportunity to explore the content in more depth, discuss important clinical issues, ask questions that may have arisen, clarify important concepts, and collaborate with international students. Students will discuss differences in culture and healthcare related to each Module. Time will be provided for students to interact with one another in breakout rooms to facilitate engagement.

#### Continued involvement in the OVC

Students will have access to the OVC until the completion of the COIL project. This will provide continued opportunities for students to engage with one another, access resources, connect with faculty, and receive updates about the project, global nursing opportunities and COIL.

#### Virtual Community of Practice (VCoP)

The VCoP will be the primary forum for faculty to collaborate on current and future research projects. This includes staff involved in the educational aspects of the COIL project and staff who are interested in engaging in international collaboration for research. It will facilitate an online nursing global community where nursing areas of interest are shared, potential collaborative research ideas and projects are created, and considerations related to the COIL project are discussed.

### Data collection

#### Students

A mixed methods approach will be utilised consisting of pre and post-test surveys and focus group interviews for both students and faculty involved in the study. Two time points will be used for data collection from the surveys that will be conducted according to key COIL project milestones. Students will be asked to complete the surveys online using Qualtrics™. Each survey will take approximately 10–15 min to complete.

A selection of consenting students will be invited to participate in a focus group or individual interview using Zoom™ video conferencing. We intend to recruit a cross section of students from each cohort, country, and course.

#### Faculty

All faculty teachers involved in facilitating the COIL teaching and learning experiences and research collaborations will be invited to complete a survey online using Qualtrics. The survey will take approximately 10–15 min to complete. A selection of consenting faculty will be invited to participate in an online focus group or individual interview using Zoom™ video conferencing. We intend to recruit a cross section of faculty from each cohort, country and course.

#### Project team

Members of the project team will be invited to participate in an online focus group or individual interview using Zoom™ video conferencing. All focus group interviews will take approximately 1 h and will be audio-recorded and transcribed.

Using pre and post-test surveys, quantitative measures will measure growth in interpersonal communication skills, adaptability, confidence, cultural awareness, perspectives on differences and similarities of nursing practices, peer collaboration and the effect of the teaching and learning interventions. Qualitative measures will focus on gaining further insight into the COIL experience from the perspective of all participants, including students, faculty, and the project team. The study will collect data at various timepoints in 2022, as outlined in Table 3.

**Table 3:** Protocol for data collection across 2022

Stage	Collection
Pre-COIL Before first VGC	**Student Survey 1 (Pre-test)** • Student characteristics • Modified International Cross Cultural Experiential Learning Survey ◦ Motivation to participate in COIL ◦ Expectations for the COIL Course
Post-COIL After final VGC for each semester	**Student Survey 2 (Post-test)** • Information of student participation in COIL interventions • Modified International Cross Cultural Experiential Learning Survey • Views on the COIL Learning Experience **Faculty Survey 1** • Impact on personal skills, cultural awareness, teaching, collaboration • Views of the COIL experience (OVC, VGC, VCoP)
Post-COIL (Students, Faculty, Project Team)	**All groups: Online FGIs** • Perceptions of the COIL experience • Perceptions of specific aspects of the project

### Instruments

Two types of instruments will be used in this study:

- Surveys
  - *Students*: The Student Pre-test Survey will gather information about student demographics and their prior experiences with travel, international activities, and COIL. A modified version of the International Cross-Cultural Experiential Learning Evaluation Toolkit [17] will be used pre and post the COIL to identify the motivators for students participating in a COIL program (7 items), their expectations of the COIL program (7 items) and self-reported views of the impact of the COIL program (7 items). Additionally, the post-test survey will seek to measure students’ desire to undertake a future student or work exchange.
  - The post-test survey will include Likert scale responses to measure students’ views on all aspects of the COIL educational experience (17 items on a 5-point scale). Open ended questions will elicit student perceptions of their overall COIL experiences.
  - *Faculty*: The faculty survey will be conducted post the COIL experience. It will include basic demographic information, prior experience with COIL, and self-reported views of the impact of the COIL program. Perceptions of specific aspects of the COIL teaching experience and the VCoP will be sought using 17 items on a 5-point Likert scale. Interviews will use a semi-structured question format. Open ended questions will elicit student perceptions of their overall COIL experiences.

- Focus group interviews
  - *Student*: Up to four semi-structured focus group interviews will be conducted with up to 10–12 students in each. Questions will be focused on the student’s views about their general learning experiences with COIL.
  - *Faculty and project team*: Semi-structured individual or focus group interviews will be conducted with faculty and the project team who were associated with development, delivery and marketing of COIL and engagement with the VCoP. Questions will be focused on the participant’s views about COIL, its contribution to student learning, the organisational impact and the level of support provided to assist with facilitation.

### Data analysis

#### Quantitative data

Using SPSS, descriptive statistics will be utilized to determine an overview picture of the data. Demographic variables will be used to gain better insight into other variables of interest. The results for all statistical assumption testing will be reported, which may affect the statistical test used for analyses. For the comparison between pre- and post- surveys, a range of parametric and non-parametric tests will be utilized, including, but not limited to t-tests and chi-squares.

#### Qualitative data

Survey data on open-ended responses will be transcribed and qualitative data will be analysed and reported thematically using Excel. Two researchers will code the data and any tenable strategies to achieve trustworthiness of data will be implemented.

### Risk management and safety

It is anticipated that there will be no physical, psychological, social, legal, or financial harm to the participants involved in this study. Participants may withdraw from the study at any time. Students will be informed that participating is voluntary and will not impact their educational standing whether they choose to participate or not. No identifying information will be included in any survey and identifying information will be redacted from focus group data.

### Data security & handling

Survey data will be collected online and saved in a password protected Qualtrics™ account, or paper-based surveys will be entered directly into Qualtrics™. Other information (consent form) will be collected and stored in the researchers’ OneDrive™, of which only the researchers have access via their institution login and password. During and following completion of the study, project documentation will be kept in a secure, lockable location in the office of one of the lead researchers. Data will be stored for 7 years. Access to data will be limited to the chief investigators and support staff only. IDs will be generated for participants to login to the CenerioVR software application, to allow them to complete the VR/VSim scenarios for the VGCs. The software company will not have access to any identifying features of individual students. Focus groups will be recorded and transcribed via auto-transcription in Zoom™ or by an administrator. These recordings will only be available to the researchers via use of a password. Any identifying information will be removed from the transcripts being analysed.

### Confidentiality and security

All survey information collected will be anonymous and no individual will be identifiable in any of the reporting of outcomes. Students will be required to enter their student number on each survey for the purposes of matching the pre and post intervention data for statistical analyses. The research team will not be privy to any names associated with this information. As the use of student ID has the potential to be re-identifiable, an independent researcher will generate another number for each participant for the data analysis. Only first names will be used in the focus group interviews and transcripts. Any information that identifies participants in this project will be removed from interview transcripts. Zoom™ administrators will transcribe the audio recordings to ensure adherence to ethical principles. Only the administrators will have access to the audio recordings and interview transcripts prior to the removal of identifying information. Findings of the research will be presented in a de-identified manner in published peer-reviewed journals and presented at conferences. It will not be possible to identify students from any information presented in this manner. During the study, all files will be kept secure for the duration of the project. Following completion of the study, project documentation will be kept in a secure, password protected location, such as OneDrive™. Data will be stored for 5 years.

## Discussion

This study aims to evaluate the design, implementation, and outcomes of a unique COIL program between Australian and Canadian nursing students and faculty. It will measure the impact of COIL as a vehicle to deepen global engagement of place bound students and faculty. It will determine whether COIL enables students and faculty to work collaboratively to address globally relevant issues, and if it facilitates the exploration of self and cultural awareness. Outcomes of the COIL program will demonstrate its ability to foster an appreciation of cultural diversity in students and promote international research collaboration and research productivity for nursing faculty. Given the infancy of research into COIL programs in the health professions, the study represents a unique example of how to co-design effective virtual international educational experiences for students using innovative digital technologies. In addition, it will expand the evidence base for COIL in nursing education and provide a research template that could be replicated in other countries and contexts.

The findings of this study will be disseminated in peer reviewed journals and via national and international conferences from within and outside the partnering institutions’ countries. We propose that our approach is an innovative, scalable, and cost-effective way to address current global health concerns and to prepare a highly capable global nursing workforce.

### Strengths and limitations

The interventions developed in this study can be used in other nursing courses and the program is a cost-effective and a scalable alternative to traditional exchange programs. There is enhanced flexibility of delivery of the interventions through both synchronous and asynchronous engagement and advanced technologies enable immersive learning experiences. The study will have some limitations such as language and terminology barriers, access and literacy of technologies, time-zone disparities, including semester timelines and funding support.

## Data Availability

The data that support the findings of this study will be available from the corresponding author, LS, upon reasonable request.

## Abbreviations

COIL: Collaborative online international learning
SBE: Simulation-based Education
VR: Virtual Reality
VSim: Virtual Simulation
VGC: Virtual Global Classroom
OVC: Online Virtual Community
VCoP: Virtual Community of Practice

## References

[1] Gakumo CA, Darwish SA, Dawson MA (2021). Preparing Students for Interprofessional Work in the Global Village: The Role of Nurse Educators. Teaching in Nursing and Role of the Educator: The Complete Guide to Best Practice in Teaching, Evaluation, and Curriculum Development.

[2] Parker V, McMillan M (2007). Challenges facing internationalisation of nursing practice, nurse education and nursing workforce in Australia. Contemp Nurse.

[3] Shishani K, Allen C, Shubnikov E, Salman K, LaPorte RE, Linkov F (2012). Nurse educators establishing new venues in global nursing education. J Prof Nurs.

[4] World Health Organization (2016). Nurse educator core competencies.

[5] Liu Y, Shirley T (2021). Without crossing a border: exploring the impact of shifting study abroad online on students' learning and intercultural competence development during the COVID-19 pandemic. Online Learning.

[6] Jung D, De Gagne JC, Choi E, Lee K (2022). An Online International Collaborative Learning Program during the COVID-19 pandemic for nursing students: mixed methods study. JMIR Med Educ.

[7] Männistö M, Mikkonen K, Vuopala E, Kuivila H-M, Virtanen M, Kyngäs H (2019). Effects of a digital educational intervention on collaborative learning in nursing education: a quasi-experimental study. Nordic J Nurs Res.

[8] Wihlborg M, Friberg E. Framework for a virtual nursing faculty and student learning collaboration between universities in Sweden and the United States: a theoretical paper. Nurse Educ Today. 2016;41:50–3. 10.1016/j.nedt.2016.03.012. 10.1016/j.nedt.2016.03.012 27138482

[9] Bragadottir H, Potter T (2019). Educating nurse leaders to think globally with international collaborative learning. Nordic J Nurs Res.

[10] Chen X, Liu S, Tao Z, Ou Y, Xiao Y (2022). Application of network teaching in nursing undergraduate education during the coronavirus disease 2019 epidemic. BMC Med Educ.

[11] State University of New York (2022). The SUNY COIL Centre.

[12] Potter T, Bragadottir H, Dyson S, McAllister M (2020). Collaborative online international learning (COIL): a new model of global education. Routledge International Handbook of Nurse Education.

[13] University of Washington| Bothell (2022). Resources for COIL Course Development.

[14] Niitsu K, Kondo A, Hua J, Dyba NA. A Case Report of Collaborative Online International Learning in Nursing and Health Studies Between the United States and Japan. Nurs Educ Perspect. 2022. 10.1097/01.NEP.0000000000000974. 10.1097/01.NEP.0000000000000974 35420569

[15] Bennett JM, Bennett MJ, Hawkins S (2004). Developing intercultural sensitivity: An integrative approach to global and domestic diversity. Handbook of Intercultural Training.

[16] ELB Learning (2022). ELB Learning.

[17] International Cross-Cultural Experiential Learning Evaluation Toolkit (2015). International Cross-Cultural Experiential Learning Evaluation Toolkit..

